# Cinnamaldehyde ameliorates obesity-induced nephropathy in C57BL/6 mice via modulation of AMPK/ACC and NF-kB pathways

**DOI:** 10.22038/ijbms.2025.83913.18157

**Published:** 2025

**Authors:** Himani Gupta, Uma Bhandari

**Affiliations:** 1 Department of Pharmacology, School of Pharmaceutical Education & Research (SPER), Jamia Hamdard, New Delhi-110062, India

**Keywords:** AMPK pathway, Cinnamaldehyde, Inflammation, NF-kB pathway, Obesity-associated – nephropathy, Oxidative stress

## Abstract

**Objective(s):**

Chronic kidney disease (CKD) is a life-threatening condition often resulting from obesity and other pathologies. The present study assesses the nephroprotective effect of Cinnamaldehyde against high-fat diet (HFD) obesity-associated nephropathy in rodents.

**Materials and Methods:**

The molecular docking analysis on AMPK & NF-kB was carried out to identify possible targets of Cinnamaldehyde. In preclinical study, 4-week-old C57BL/6 mice (18–20 gm) were fed a conventional diet or HFD for 12 weeks After the fifth week of HFD intervention, mice were divided into six groups (n=10): vehicle group; HFD group; HFD+CA (20 mg/kg); HFD+CA (40 mg/kg); HFD+Orlistat (10 mg/kg); and CA Perse (40 mg/kg) treated orally for 49 days. On day 84, mice were fasted overnight, and urine and blood were collected for various biochemical analyses. Animals were sacrificed, and kidneys were removed for histopathology and immunohistochemistry.

**Results:**

*In silico* studies showed strong binding of Cinnamaldehyde with AMPK and NF-kB. Cinnamaldehyde showed a significant (*P*<0.001) decrease in BW, BMI, blood glucose, leptin, insulin, HOMA-IR, total cholesterol, triglycerides, creatinine, albumin, TNF-α, IL-6, and IL-β in serum and urinary albumin. It also produced a significant (*P*<0.001) reduction in KIM-1, type-IV collagen, IL-18, and NGAL urinary levels. Further, it produced a significant (P<0.001) increase in urine creatinine, serum adiponectin, and kidney SOD, GSH, GST, and GPx. Immunohistology indicated suppressed NF-kB and activated AMPK/ACC pathways. Histopathology showed improvement in glomerular inflammation, tubular injury, and degeneration in kidney tissue.

**Conclusion:**

Cinnamaldehyde significantly protects against obesity-associated nephropathy in C57BL/6 mice by HFD via modulating the AMPK/ACC and NF-kB pathways.

## Introduction

Metabolic changes significantly affect health due to their apparent role in regulating homeostasis in the body. Recent trends in disease progression indicate a steep rise in diseases of metabolic origin. These include diabetes, thyroid dysfunction, insulin resistance, dysregulated fat metabolism, and obesity (1). 

Excess in the quantity and distribution of body fat is generally identified as obesity. Excessive visceral fat is concerning as it causes other health issues (1). Obesity poses significant health problems like cardiovascular issues and kidney damage. Therefore, the problem needs to be addressed globally. Obesity is dramatically related to lifestyle, as it occurs when there is a lack of exercise, excess consumption of unhealthy food, and lack of mobility, which slow down the metabolic rate in individuals (1). Additionally, changes in dietary habits, including increased consumption of fat-rich diets and calorie-dense foods, have further aided the rise in excess weight and obesity (2, 3).

The kidney, a major metabolizing organ, is highly vulnerable to obesity. Renal disorders result from metabolic disorders, specifically insulin resistance, dysfunctional fats, and carbohydrate metabolism (1). Obesity and nephropathy are intricately associated due to their complex physiologies. Obesity-induced nephropathy is marked by fundamental and architectural alterations of the kidney that result in compensated renal functioning (1, 2). The last two decades have witnessed about 1.9 billion overweight adults on the global platform, while obese adults make up 650 million (4).

Obesity causes hyperfiltration of the glomerular system and tubular vasodilation to regulate the ionic balance regardless of increased tubular reabsorption (5). These compensatory mechanisms potentially injure the glomerulus over time, which then intensifies hypertension and further damages the kidneys. This develops a vicious cycle that is triggered by elevated arterial pressure, metabolic abnormalities, and glomerular injury (5). In addition, elevation in inflammatory biomarkers *via *Tumour necrosis factor-α (TNF-α) and Interleukins (ILs) also critically regulates the development of obesity-induced nephropathy (5). 

Kidney damage is an irreversible process that develops chronic kidney disease (CKD) and subsequently demands hemodialysis and renal transplantation to increase life expectancy. The advanced stage of obesity-induced nephropathy is known as CKD. Therefore, it is essential to manage body weight and obesity for a healthier existence. Even though obesity is largely preventable with lifestyle changes, sometimes medical intervention is desired to prevent other complications like nephropathy (5). A variety of oral drugs are available for the medical intervention of obesity and obesity-associated nephropathy, such as dipeptidyl peptidase 4 inhibitor (DPP-4), angiotensin-converting enzyme (ACE) inhibitors, GLP-1(Glucagon-like-1) protein receptor agonists, sodium-glucose cotransporter 2 inhibitors (SGLT-2), and statins (5). It is difficult to sustain existing treatments over the long term because they have several side effects, like myopathy (GLP-1 receptor agonists), pancreatitis (DPP-4 inhibitors), **urinary tract infections **and **genital infections** (SGLT-2), bladder cancer, angioedema (ACE inhibitors), etc.; further, these are not cost-effective, and GLP-1 receptor agonists are orally inactive. Recently, investigators have pursued herbal therapy regimens that can treat various ailments cost-effectively and have comparatively fewer or no side effects. Therefore, it necessitates identifying novel formulas and therapeutic techniques that are both safe and effective.

High-fat diet (HFD)-induced obesity is one of the most popular, reliable, reproducible, and effective experimental models for evaluating obesity and related pathologies like nephropathy (6). In animal models, HFD induces clinical and histological changes in renal tissues that closely mimic obesity-induced nephropathy observed in humans. Long-term administration of HFD in mice triggers the production of cytokines and chemokines, leading to renal dysfunction and damage (7). Medicinal agents from plant, herbal, and natural sources have seized immense attention in recent drug development. The natural compounds obtained from leaves, roots, barks, etc., contain various essential phytonutrients that can manage dysregulated metabolic activity and aid metabolism. One such compound is Cinnamaldehyde. Cinnamaldehyde (CA), a distinctive compound derived from the bark of *Cinnamomum cassia* (cinnamon), is extensively utilized in pharmaceuticals, food products, and spices. The FDA has designated CA as “Generally Recognized as Safe (8).” It is an herbal remedy used to cure disorders, including diabetes, obesity, antibacterials, and non-alcoholic fatty liver disease (NAFLD). Its antidiabetic and obesity potential has been found in animal models by some researchers of obesity and diabetes (8). Moreover, several researchers have thoroughly examined the protective effects of cinnamon on the kidneys (9,10). Nevertheless, research on the phytoconstituents accountable for their nephroprotective efficacy is still lacking. 

The current investigation was designed to investigate the possibility of Cinnamaldehyde in ameliorating obesity-associated nephropathy. In this study, we aimed to assess the *in silico* binding capacity of Cinnamaldehyde with AMP-activated protein kinase (AMPK) and nuclear factor kappa-light-chain-enhancer of activated B cell (NF-kB). This was followed by a preclinical evaluation of Cinnamaldehyde in obesity-associated nephropathy in rodent models. To the best of our information, this is a novel study that explores the affinity of Cinnamaldehyde with AMPK and NF-kB and its preventive effect in HFD-induced obesity-associated nephropathy. 

## Materials and Methods

### Drugs and chemicals

The National Institute of Nutrition (NIN) in Hyderabad supplied HFD. Cinnamaldehyde was purchased from Yucca Enterprises, Mumbai, India. Orlistat was purchased from a local chemist shop in New Delhi, India. The Accu-Check glucometer was acquired from Roche Diabetes Care India Pvt. Ltd., Mumbai, Maharashtra, India. Lipid Kits were procured from Span Diagnostic Ltd., Surat, India. ELISA kits were purchased from Krishgen Biosystem, Mumbai, Maharashtra (Creatinine, albumin, leptin, adiponectin, and insulin), India and ELK Biotechnology Co., Ltd., Denver, USA (KIM-1, TNF-α, IL-6, and IL-β type-IV collagen).

### In silico analysis

**Molecular docking was analyzed using AutoDock Vina 1.5.7 to identify the ligand binding site of AMPK and NF-kB. **The RCSB Protein Data Bank was used to determine the three-dimensional X-ray crystallographic structures of NF-kB (PDB ID: 40T9) and AMPK (PDB ID: 4CFF). The ligand structures of orlistat and Cinnamaldehyde were obtained from the database PubChem. The Open Babel Program Version 3.1.1 was used to perform the ligand structures from Chem Sketch to PBD format to prepare proteins. The Biovia Discovery Studio Visualizer was used to import each protein separately. Polar atoms of hydrogen were added, and the attached molecules and co-crystal ligands were taken out. In AutoDock 2.0, the receptors and proteins were stored in pdbqt format. The grid dimensions were 109.88Å, 114.68Å, and 101.66Å for the x, y, and z axes. The grid center with -29.48, -12.46, and 175.38 was employed to figure out the binding site of AMPK. To determine the binding site for NF-kB, a grid box with dimensions of 85.09Å, 61.01Å, and 70.91Å for x, y, and z, respectively, and a grid box center of 25.43, 73.89, and -9.53 were created. Protein’s exhaustiveness was set to 100. Dock attitude was preserved as the optimal conformation with the least amount of energy was selected. The Discovery Studio visualizer was used to generate the ligand interaction diagram and the docked pose.

Docking scores were derived from a scoring function that considers various factors, including van der Waals forces, hydrogen bonding, and desolvation effects. Our analysis ranked the ligands based on their dock scores to identify the most promising candidates for further experimental validation. Additionally, we performed a post-docking analysis, visualizing ligand-protein interactions using tools like BioVia Visualizer to examine key interactions such as hydrogen bonds and hydrophobic contacts. We also compared our dock scores with available experimental data to validate our predictions, reinforcing the reliability of our findings. This comprehensive approach quantifies ligand efficacy and enhances our understanding of ligand-protein interactions, guiding us in prioritizing compounds for testing**.**

### In vivo studies 

#### Animals

We procured four-week-old C57BL/6 mice (18–20 gm) from Jamia Hamdard’s Central Animal House Facility in New Delhi. They were housed in well-ventilated polypropylene cages in an accredited animal house facility with a 12-hour cycle of light and dark, 25±2 °C temperature, and humidity of 55–60%, and the mice were provided with unlimited food and water. Before the feeding intervention, the mice were given one week to get used to the experimental environment. The study adhered to the Committee for Control and Supervision of Experiments on Animals (CPCSEA) guidelines. The study procedures received permission from the Institutional Animal Ethical Committee (IAEC) at Jamia Hamdard in New Delhi (Protocol No. 1972 dated 21/12/2022).

### Induction of HFD-induced obesity-associated nephropathy

HFD-induced obesity is a widely used model to evaluate obesity-associated nephropathy. This model mimics several features of human obesity and its metabolic complications. HFD provides higher calories from fats (40–60%) and reduces the amount of carbohydrates (15–25%) and protein (15–20%). It includes fat sources such as animal fat (lard), coconut oil, groundnut oil, and soybean oil. The carbohydrate sources are corn starch and sucrose, while the protein sources include casein and soy. HFD induces nephropathy by increasing lipotoxicity, oxidative stress, inflammation, and endoplasmic reticulum stress. 

The doses of Cinnamaldehyde were chosen based on available reports (11). The study was carried out for 12 weeks, and animals were housed in their cages, divided into six groups (n=10), and fed on a diet regimen as mentioned below. Groups II, III, IV, and V received HFD, and Groups I and VI received the normal diet for 12 weeks. From the sixth week, carboxymethylcellulose, Cinnamaldehyde, and Orlistat were given orally in the respective groups till the end of the experiment. Daily food and water intake were measured for the whole experimental duration, i.e., 12 weeks. 

Group I: Vehicle Group: normal diet + Vehicle (0.5% carboxymethylcellulose, PO) 

Group II: HFD Group: HFD + Vehicle (0.5% carboxymethyl cellulose, PO) 

Group III: HFD + Cinnamaldehyde (20 mg/kg, PO) 

Group IV: HFD + Cinnamaldehyde (40 mg/kg, PO)

Group V: HFD + Orlistat (10 mg/kg, PO) 

Group VI: normal diet + Cinnamaldehyde *Perse* (40 mg/kg, PO) 

### Sample collection

Urine samples were taken after being housed in separate metabolic cages for the duration of the experiment. On the 84^th^ day, animals fasted the entire night; blood was collected by puncturing the eye’s retro-orbital plexus (ROP) under ophthalmic anesthesia (1–2 drops Procainamide hydrochloride, 0.05%). The collected blood was centrifuged for 10 min at 4000 rpm/min for serum separation. The serum was refrigerated at -80 °C to assess the biochemical parameters. Immediately after blood collection, animals were euthanized under mild anesthesia by carbon dioxide (CO_2_), the kidneys of the animals were removed, and their weight was measured. 

### Assessment of anthropometric parameters

The body weight of the animals was measured weekly for 12 weeks. The change between initial and final weight was tracked to calculate weight gain. The formula was used to compute the body mass index (BMI).

BMI = body weight (gm)/length (cm^2^) (12).

### Measurement of blood glucose

The Accu-Check glucometer was used to determine the blood glucose level of the blood samples.

### Estimation of serum insulin and insulin resistance (IR)

The ELISA Mouse Insulin Kit was used to quantify serum insulin levels, following the manufacturer’s recommendations (Krishgen Biosystem, Mumbai, Maharashtra, India). IIR was estimated using the homeostasis model of assessment (HOMA-IR).

HOMA IR: blood glucose (mg/dl) × serum insulin (IU/ml)/405 (13).

### Determination of adipocytokines in the serum

Serum leptin and adiponectin levels were analyzed using ELISA Mouse leptin and adiponectin in compliance with the manufacturer’s recommendations (Krishgen Biosystem, Mumbai, Maharashtra, India).

### Determination of serum lipid level

Measuring the serum lipid levels (Total triglycerides and Total cholesterol) using a commercially available kit (Span Diagnostic Ltd., Surat, India) by following the procedure mentioned by the manufacturer.

### Determination of proinflammatory cytokines

Serum TNF-α, IL-6, and IL-β were analyzed using ELISA kits for mice, as per the manufacturer’s instructions (ELK Biotechnology Co., Ltd., Denver, USA).

### Estimation of creatinine, albumin, and BUN

Serum and urine creatinine and albumin levels were analyzed using an ELISA for mouse creatinine and albumin in compliance with the manufacturer’s procedure (Krishgen Biosystem, Mumbai, Maharashtra, India). Blood urea nitrogen (BUN) in serum was estimated using an available colorimetry kit.

### Estimation of urinary biomarkers

Urinary KIM-1 (Kidney Injury Molecule-1), Type IV collagen, neutrophilic gelatinase-associated lipocalin/lipocalin-2 (NGAL), and IL-18 levels were estimated using ELISA kits as per the manufacturer’s instructions (ELK Biotechnology Co., Ltd., Denver, USA).

### Estimation of anti-oxidants enzymes in kidney tissue

Various anti-oxidant parameters were analyzed in kidney tissue. The superoxide dismutase (SOD) concentrations were determined using Marklund & Marklund’s methodology (1974) (14). Reduced glutathione (GSH) and Glutathione peroxidase (GPx) were determined using methodology by Sedlak and Lindsay, 1968 (15), and glutathione s-transferase (GST) was determined using Habig *et al*., 1974 (16).

### Histopathology examination

**Kidneys were removed at the end of the study**, encased in paraffin, and preserved with a 4% buffered paraformaldehyde solution. Paraffin sections 3–4 mm thick were dewaxed and introduced to water using graded ethanol. Hematoxylin-eosin stain (H&E) was applied to the sections, followed by graded ethanol dehydration, xylene clearing, and distyrene xylene mounting. Under a light microscope (Meiji microscope, Japan), H&E-stained sections were graded to confirm the morphological assessment of renal tissue injury. The 40x magnification was used to take the photomicrographs.

### Immunohistochemical analysis of kidney 

Kidney sections were first stained with primary antibodies against AMPK and NF-kB at 4 °C for an entire night. Then, they were treated with Horseradish peroxidase (HRP)-labeled secondary antibodies for 30 min at 37 °C for immunohistochemical labeling. Following a Phosphate buffer saline (PBS) rinse, the slides were developed for five minutes using 3,3’-diaminobenzidine (DAB) and H2O2. After that, these sections were counterstained for one minute with hematoxylin. The expression levels of AMPK and NF-kB were investigated by measuring the brown staining of kidney tissue using light microscopy. The 40x magnification was used to take the photomicrographs.

### Statistical analysis

**All the values were given as the mean **± SEM. All variates were examined using a one-way ANOVA, followed by Tukey’s multiple comparisons test. If *P*<0.05, the statistics were considered significant. A program known as Graph Pad Prism 3.0 (Graph Pad Software, San Diego, California, USA) was used to analyze statistical analysis.

## Results

### In silico effects of cinnamaldehyde on AMPK and NF-kB

Table 1 depicts the dock scores of Cinnamaldehyde and Orlistat. The outcome of molecular docking depicts that Cinnamaldehyde (Dock score: -4.4) showed significant interactions during binding with the ligand-binding domain of AMPK, which is close to the Orlistat (Dock score: -5.5) interaction with the ligand-binding domain of AMPK. 

Furthermore, the interaction of Cinnamaldehyde (Dock score: -3.8) with NF-kB is near to Orlistat (Dock score: -4.6)

**Figure 1:**
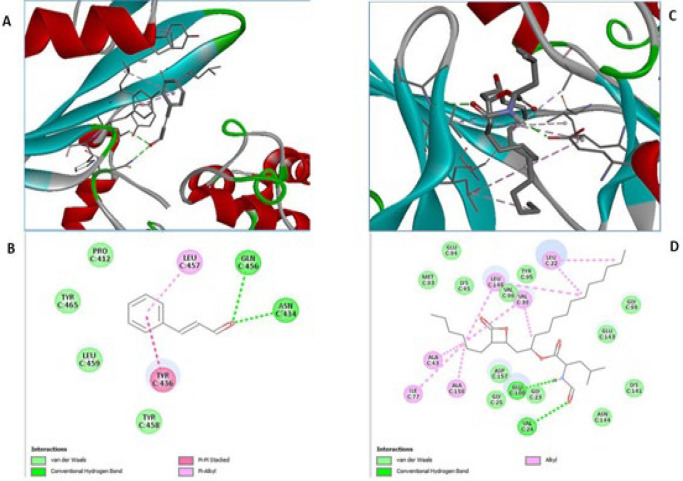
Binding mode and ligand interaction diagram of Cinnamaldehyde (A), (B) and Orlistat (C), (D) in the catalytic pocket of AMPK (PDB ID: 4CFF)

The Aldehyde group of Cinnamaldehyde interacts with the AMPK receptor and forms conventional hydrogen bonds with the amide side chain of asparagine at position C:434 and glutamine at position C:456. The aromatic ring of Cinnamaldehyde interacts through pi-pi stacked interaction with tyrosine residue at position C:436. Also, this aromatic ring interacts with side chain residue leucine at position C:457 by pi-alkyl interaction. The Cinnamaldehyde molecule stabilizes itself by van der Waal force with a proline residue at position C:412, tyrosine residue at position C:456, C:458, and leucine residue at position C:458 (Figure 1). 

**Figure 2:**
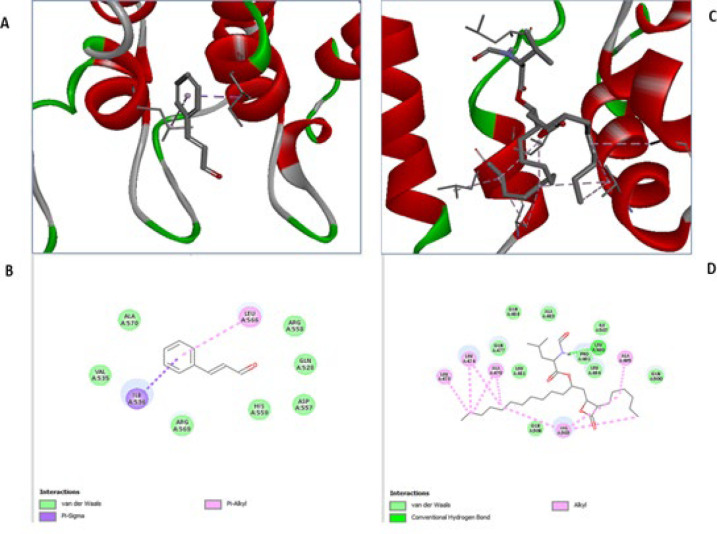
Binding mode and ligand interaction diagram of Cinnamaldehyde (A), (B) and Orlistat (C), (D) in the catalytic pocket of NF-kB (PDB ID: 4OT9)

In the case of NF-kB, the aromatic ring of Cinnamaldehyde interacts with the side chain of isoleucine at position A:536 through PI-sigma interaction. It also interacts with the side chain of leucine at position A:566 through Pi-alkyl interaction. Cinnamaldehyde stabilized by van der Waals forces with alanine at position A:570, valine at position A:535, arginine at position A:569, A:558, histidine at position A:559, aspartic acid at position A:557, and glutamine at position A:528 (Figure 2). 

Interestingly, the dock score of Cinnamaldehyde was comparable to orlistat with both receptor proteins, AMPK and NF-kB, indicating that Cinnamaldehyde can be a promising molecule for treating obesity-associated nephropathy. The 2D and 3D interaction images of Cinnamaldehyde with AMPK and NF-kB are presented in Figures 1 and 2, respectively.

### Effect of cinnamaldehyde on HFD-induced alteration in body weight (BW) and BMI

There was a notable increase (*P*<0.001) in BW in the HFD group compared to the vehicle group. Treatment with orlistat (10 mg/kg) remarkably (*P*<0.001) lowered BW compared to the HFD group. Additionally, conversely with the HFD group, Cinnamaldehyde (20 and 40 mg/kg) also significantly (*P*<0.001) reduced BW. Also, there were not any noteworthy (*P*>0.05) changes in body weight in the Cinnamaldehyde Perse (40 mg/kg) compared to the vehicle group. 

Moreover, a rise in BMI was found notably (*P*<0.001) in the HFD group compared to the vehicle group. Cinnamaldehyde (20 mg/kg) produced no significant (*P*>0.05) changes in BMI, and Cinnamaldehyde (40 mg/kg) produced a remarkable (*P*<0.001) depletion in BMI when compared to the HFD group; this is comparable with Orlistat (10 mg/kg) treatment. However, a non-significant (*P*>0.05) change was seen in the BMI of the Cinnamaldehyde perse (40 mg/kg) compared to the vehicle group (Table 2).

### Effect of cinnamaldehyde on HFD-induced alteration in food intake

It was observed that there were no noteworthy (*P*>0.05) changes in food intake in the HFD group when compared to the vehicle group. Cinnamaldehyde (20 and 40 mg/kg) reveals no changes (*P*>0.05) in food intake compared to the HFD group. In addition, orlistat (10 mg/kg) also showed results similar to those of the Cinnamaldehyde group. Also, Cinnamaldehyde perse (40 mg/kg) produced non-significant (*P*>0.05) changes in food intake (Table 2).

### Effect of cinnamaldehyde on HFD-induced alteration kidney weight and kidney/body weight

The HFD group showed a significant (*P*<0.001) rise in kidney weight vs the vehicle group. Whereas Cinnamaldehyde (20 mg/kg) showed a non-significant (*P*>0.05) change in kidney weight in comparison with the HFD group. On the other hand, Cinnamaldehyde (40 mg/kg) showed a pronounced (*P*<0.001) decrease in kidney weight when compared with the HFD group, which is similar to (*P*<0.001) Orlistat (10 mg/kg). Also, no change (*P*>0.05) was seen in the Cinnamaldehyde *Perse* (40 mg/kg) vs the vehicle group (Table 2).

Furthermore, the HFD group showed a non-significant (*P*>0.05) change in kidney/body weight compared to the vehicle group. Cinnamaldehyde (20 and 40 mg/kg) reveals no changes (*P*>0.05) in kidney/body weight compared to the HFD group. In addition, orlistat (10 mg/kg) also showed results similar to those of the Cinnamaldehyde group. Also, Cinnamaldehyde perse (40 mg/kg) produced non-significant (*P*>0.05) changes in kidney/body weight (Table 2). 

### Effect of cinnamaldehyde on HFD-induced changes blood glucose, serum insulin, and IR

Blood glucose, serum insulin, and HOMA-IR levels were prominently elevated (*P*<0.001) in the HFD compared to the vehicle group. Elevated blood glucose and serum insulin levels and IR were significantly decreased (*P*<0.001) by orlistat (10 mg/kg) in contrast to the HFD group. Cinnamaldehyde (20 mg/kg) also markedly decreased the blood glucose (*P*<0.05), serum insulin, and IR (*P*<0.001) vs the HFD group. However, HFD-induced alterations in the above parameters were more effectively (*P*<0.001) reduced by higher Cinnamaldehyde (40 mg/kg) compared to the HFD group. No significant modulation in the above parameters was observed in the Cinnamaldehyde* Perse* (40 mg/kg) group (Table 3). 

### Effect of cinnamaldehyde on HFD-induced alteration of lipid levels

**The group fed with HFD exhibited a notable rise in TG and TC (** *P*<0.0**01) levels compared to the vehicle group. Cinnamaldehyde (20 mg/kg) remarkably reduces TG **(*P*<0.05) and TC (*P*<0.01) levels compared to the HFD group**. Treatment with a **higher dose of Cinnamaldehyde (40 mg/kg) showed a pronounced decrease in TG and TC (*P*<0.001) compared to the HFD group, with outcomes comparable to those achieved with orlistat (10 mg/kg) (*P*<0.001). However, no visible changes (*P*>0.05) were found in the Cinnamaldehyde perse (40 mg/kg) group (Table 4).

### Effect of cinnamaldehyde on HFD-induced changes in BUN, serum creatinine, and serum albumin

HFD for 12 weeks caused a remarkable rise in BUN, serum creatinine, and serum albumin levels (*P*<0.001) compared with the vehicle group. Cinnamaldehyde (20 mg/kg) treatment revealed a remarkable (*P*<0.05) decrease in serum creatinine, serum albumin, and BUN (*P*<0.01) in comparison with the HFD group. In contrast, Cinnamaldehyde (40 mg/kg) showed a higher significance decrease in BUN, serum creatinine, and albumin levels (*P*<0.001). These results were similar to the Orlistat (10 mg/kg) treated group (*P*<0.001). Cinnamaldehyde perse (40 mg/kg) showed no remarkable change in the above parameters in the comparison vehicle group (Table 5). 

### Effect of cinnamaldehyde on HFD-induced changes in adipocytokines

**Figure 3:**
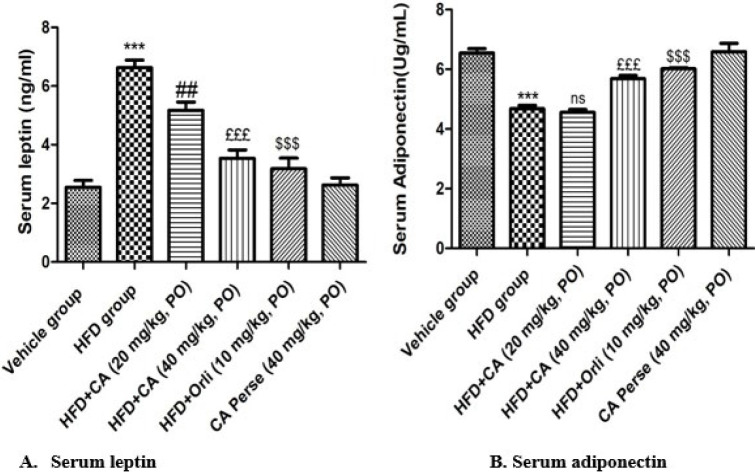
Effects of Cinnamaldehyde on serum leptin and serum adiponectin levels in HFD-induced obesity-associated nephropathy in C57BL/6 mice

Serum leptin was significantly elevated, while serum adiponectin was significantly decreased (*P*<0.001) in HFD vs the vehicle group. Orlistat (10 mg/kg) substantially decreases leptin levels and significantly increases (*P*<0.001) adiponectin compared to the HFD group. Cinnamaldehyde (20 mg/kg) also markedly attenuated serum leptin (*P*<0.01) but showed no noteworthy (*P*>0.05) changes in serum adiponectin level when compared to the HFD-fed mice. HFD-induced alterations in leptin and adiponectin were more efficiently (*P*<0.001) suppressed by Cinnamaldehyde (40 mg/kg) compared with the HFD group. However, no significant (*P*>0.05) changes were observed in the Cinnamaldehyde perse (40 mg/kg) group in comparison to the vehicle group (Figure 3 A and B).

### Effect of cinnamaldehyde on HFD-induced changes in urinary parameters

**Figure 4:**
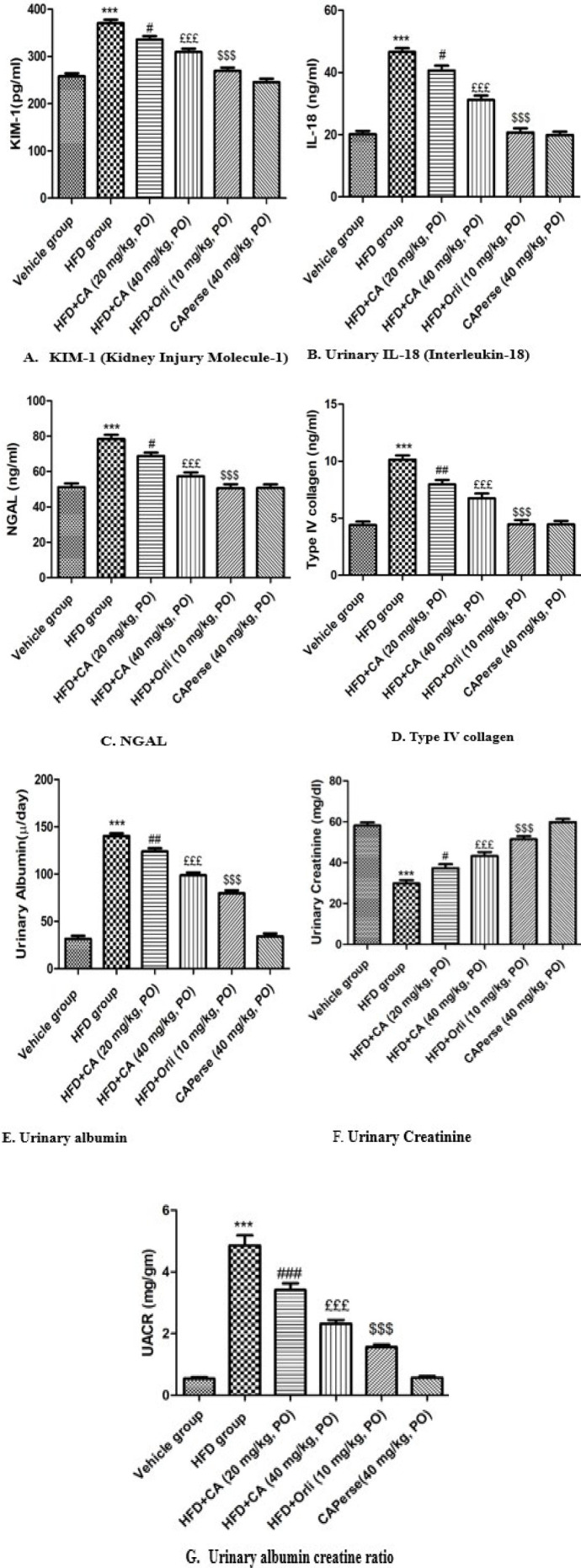
Effects of Cinnamaldehyde on KIM-1, urinary IL-18, NGAL, type-IV collagen, urinary albumin, urinary creatinine, and urinary albumin creatinine ratio in HFD-induced obesity-associated nephropathy in C57BL/6 mice

Administration of HFD for 12 weeks led to a significant (*P*<0.001) elevation in urinary albumin, urinary albumin creatinine ratio, IL-18, KIM-1, Type IV collagen, and NGAL levels compared to the vehicle group. In contrast, urinary creatinine levels reduced noticeably (*P*<0.001) in the HFD group. Orlistat (10 mg/kg) remarkably attenuated (*P*<0.001) HFD-induced changes in urinary markers compared to the HFD group. Additionally, Cinnamaldehyde (20 mg/kg) significantly decreased (*P*<0.05) urinary IL-18, KIM-1, NGAL, urinary albumin, type IV collagen (*P*<0.01), and urinary albumin creatinine ratio (*P*<0.001). Whereas urinary creatinine was notably (*P*<0.05) increased compared to the HFD. HFD-induced changes were more remarkably (*P*<0.001) attenuated by Cinnamaldehyde (40 mg/kg) in comparison with the HFD group. The Cinnamaldehyde *Perse* (40 mg/kg) group showed no notable difference in urinary biochemical parameters compared to the vehicle group (Figure 4 A to G).

### Effect of cinnamaldehyde on HFD-induced changes in serum proinflammatory cytokines

**Figure 5:**
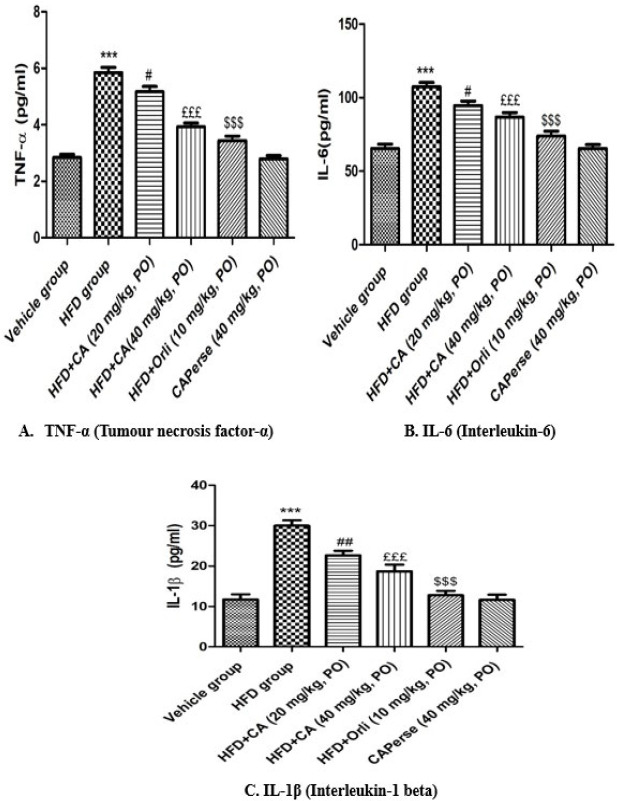
Effect of Cinnamaldehyde on serum TNF-α, IL-6, and IL-1β levels in HFD-induced obesity-associated nephropathy in C57BL/6 mice

HFD showed a remarkable increase (*P*<0.001) in the levels of serum proinflammatory cytokines *via *TNF-α, IL-6, and IL-1β compared with the vehicle group. Cinnamaldehyde (20 mg/kg) significantly reduced the levels of (*P*<0.05) TNF-α, IL-6, and (*P*<0.01) IL-1β compared to the HFD group. On the other hand, Cinnamaldehyde (40 mg/kg) produced a more (*P*<0.001) significant reduction in the levels of above serum proinflammatory cytokines in comparison with the HFD group; this is comparable to the orlistat (10 mg/kg) group. However, there was no discernible variation in the amounts of these serum proinflammatory cytokines for Cinnamaldehyde perse (40 mg/kg) compared to the vehicle group (Figure 5 A to C).

### Effects of cinnamaldehyde on HFD-induced alterations on oxidative stress

**Figure 6:**
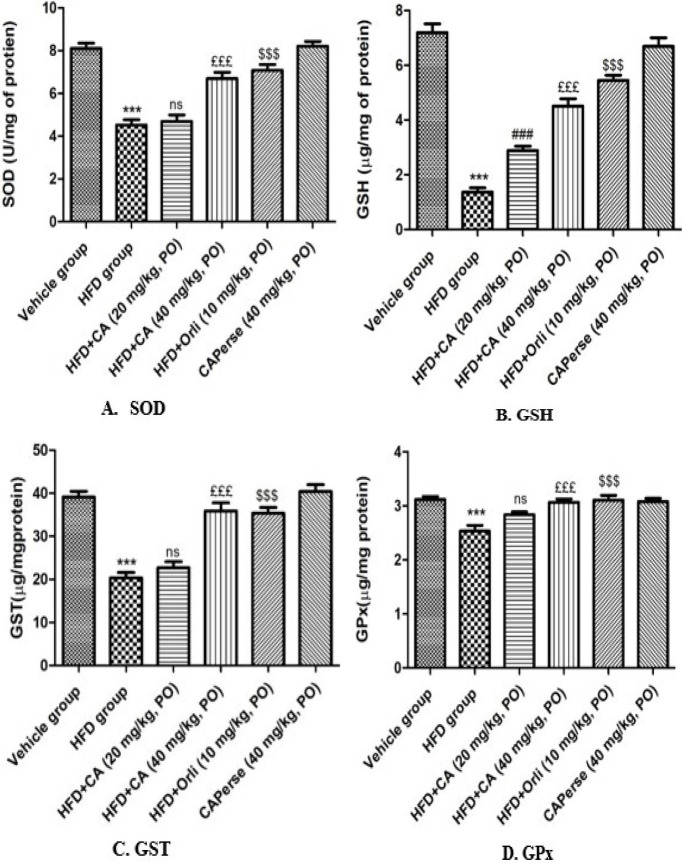
Effect of Cinnamaldehyde on kidney’s SOD, GSH, GST, and GPx levels in HFD-induced obesity-associated nephropathy in C57BL/6 mice

HFD remarkably (*P*<0.001) reduced the concentration of SOD, GSH, GST, and GPx when compared with the vehicle group. Cinnamaldehyde (20 mg/kg) demonstrated a non-significant (*P*>0.05) change in levels of SOD, GST, and GPx and a notable (*P*<0.001) rise in GSH level compared to the HFD group. Whereas Cinnamaldehyde (40 mg/kg) produced a more remarkable (*P*<0.001) increase in the above parameters compared to the HFD group, this is comparable to the Orlistat (10 mg/kg) group. However, there was no discernible variation in the amounts of anti-oxidants for Cinnamaldehyde perse (40 mg/kg) compared to the vehicle group (Figure 6 A to D).

### Effect of cinnamaldehyde on HFD-induced changes in renal histology

**Figure 7:**
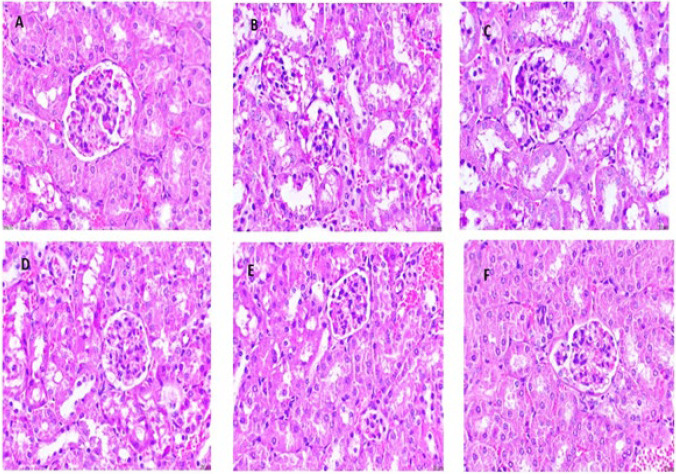
Haematoxylin and eosin (H&E) staining of Kidney tissue

The HFD-treated group exhibited histological aberration in renal tissue. The changes include glomerular inflammation (congested glomeruli), tubular injury, and tubular degeneration and necrosis (tubular cells swollen with vacuoles). Additionally, inflammatory infiltration was present in tubules and glomeruli. The vehicle group revealed normal architecture devoid of histological aberrations in the HFD-treated group. Cinnamaldehyde (20 mg/kg) treatment has shown a significant decrease in histological aberration of renal tissue. Whereas Cinnamaldehyde (40 mg/kg) and Orlistat (10 mg/kg) treatment largely attenuated HFD-induced renal damage. The Cinnamaldehyde Perse (40 mg/kg) group produced a similar architecture to the vehicle group (Figure 7 A to F). 

### Effect of cinnamaldehyde on the IHC of AMPK and NF-kB markers in kidney tissues

Immunohistochemistry of the vehicle group showed positive staining for AMPK stimulation. On the other hand, NF-kB activity in renal tissue was found to be absent. HFD treatment caused a decrease in the accumulation of immune-stained brown hue AMPK. It increased the number of NF-kB-positive cells, indicating the deactivation of the AMPK pathway and activation of the NF-kB pathway. 

**Figure 8:**
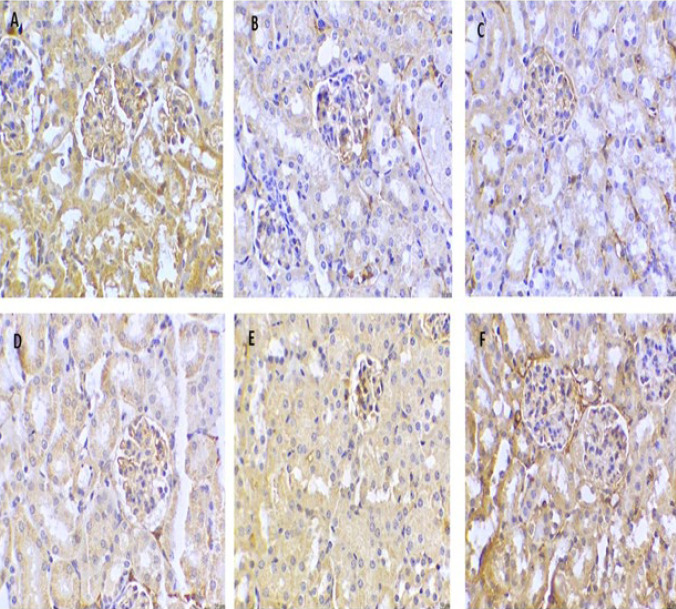
Immunohistochemistry of AMPK in Kidney tissue

**Figure 9:**
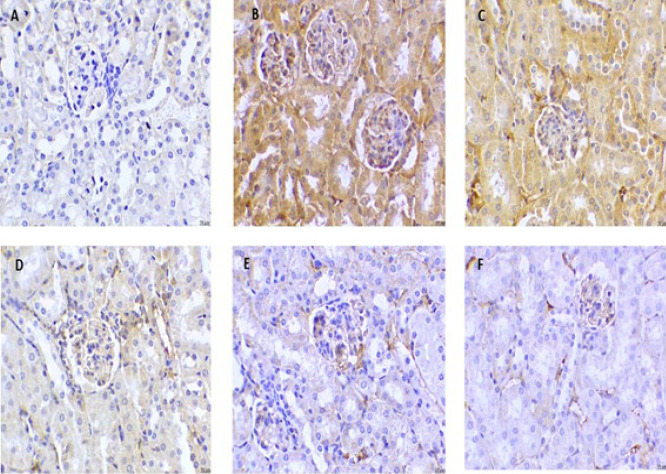
Immunohistochemistry of NF-kB in Kidney tissue

Cinnamaldehyde (20 mg/kg) led to a higher immune-stained AMPK and a minor decrease in NF-kB immune-stained positive cells compared with HFD mice. Conversely, Cinnamaldehyde (40 mg/kg) and Orlistat (10 mg/kg) treatment produced a further rise in the expression of AMPK and a decrease in NF-kB-stimulated positive cells compared with HFD mice. There were no changes in the Cinnamaldehyde Perse (40 mg/kg) group compared to the vehicle group (Figures 8 and 9 A to F).

## Discussion

The current investigation was designed to demonstrate the nephroprotective capacity of Cinnamaldehyde in obesity-associated nephropathy. Cinnamaldehyde was found to ameliorate renal changes, in addition to decreasing the serum and urinary biomarkers of renal damage, improving oxidative stress, and reducing inflammatory cytokines in HFD-induced obesity-associated nephropathy. 

Obesity is a long-term condition marked by an overpowering or excessive accumulation of body fat or adipose tissue, which can be dangerous to health. It poses an independent threat to the development and progression of nephropathy (17,18). Obesity-associated nephropathy is a growing health concern worldwide, affecting people of all ages and races (19). Pharmacological intervention for obesity-associated nephropathy primarily focuses on managing obesity, hypertension, diabetes, and dyslipidemia to protect kidney function and mitigate further damage (20). However, insight into natural products for the management of obesity-associated nephropathy has gained attention due to the unwanted side effects and high cost of therapy of synthetic medicines. So, the present study was designed to identify a nephroprotective molecule for obesity-associated nephropathy. This novel study investigated the nephroprotective potential of Cinnamaldehyde in obesity-associated nephropathy in the HFD rodent model.

Initially, we carried out molecular docking analyses to identify the attachment of Cinnamaldehyde and Orlistat into ligand-binding domains of AMPK and NF-kB. Molecular docking analysis helps determine the lead compound’s binding strength, affinity, and optimal orientation for the catalytic binding site of a target receptor (21). The AMP-activated protein kinase (AMPK) pathway is an essential energy sensor that controls the metabolism balance. AMPK is essential for maintaining metabolic health, and medicines addressing obesity, type 2 diabetes mellitus (T2DM), cardiovascular disease (CVD), and renal illness can target it to improve energy control and lower inflammation (22). The NF-kB pathway regulates immune and inflammatory response by activating genes involved in inflammation, cell survival, and proliferation (22). 

*In silico* study of orlistat showed a binding score of -5.5 and -4.6 with AMPK and NF-kB, respectively. Cinnamaldehyde revealed binding with AMPK and NF-kB with dock scores of -4.4 and -3.8, respectively. Chronic activation can lead to diseases such as obesity-associated nephropathy. Targeting this pathway therapeutically by inhibiting NF-κB activity helps reduce inflammation and tissue damage. Thus, the *in silico* analysis indicated that Cinnamaldehyde could improve inflammation through the previously unproven participation of AMPK and NF-κB proteins. As far as we are aware, we are the first to perform *in silico* binding affinity of Cinnamaldehyde with AMPK and NF-kB pathways. Further, *in vivo* studies were done to explore the role of Cinnamaldehyde in obesity-associated nephropathy *via *modulation of AMPK and NF-kB pathways.

Previous studies have presented a linear relationship between consuming a fat-rich diet and gaining weight (23). Previous reports stipulated that HFD causes obesity, as evidenced by a noticeable growth in body weight and BMI (24). In the present study, 12 weeks of administration of HFD led to a significant rise in body weight and BMI despite altering water and food intake, consistent with findings from earlier research (24).

Several biochemical parameters were assessed in serum, urine, and renal tissue to confirm the beneficial influence of Cinnamaldehyde in obesity-associated nephropathy. These biomarkers included serum insulin, blood glucose, leptin, adiponectin, creatinine, albumin, triglyceride, total cholesterol, NGAL, KIM-1 type-IV collagen, TNF-α, IL-18, and IL-β. IL-6 has a significant role in nephropathy. 

Adipocytes generate the hormone leptin, which influences the hypothalamus to control hunger and energy expenditure (25). Many studies have demonstrated that HFD induces hyperleptinemia before hyperinsulinemia (26). Evidence suggests that leptin levels are elevated in obesity and kidney injury (27). Higher leptin level stimulates reactive oxygen species (ROS) production, inflammation, and renal endothelial dysfunction. Also, higher serum leptin levels have been connected to renal hypertension and subsequent glomerular harm (28). In the present work, HFD-induced obesity-associated nephropathy caused hyperleptinemia, and Cinnamaldehyde significantly reversed the elevated leptin level to preserve kidney function. 

Adipocytes release adiponectin, a protein hormone, and adipokine, which is considered an anti-inflammatory cytokine (29). It has been reported that chronic HFD administration causes a reduction in the level of adiponectin (29). Adiponectin has anti-inflammatory and anti-oxidant properties. Collective evidence suggests reduced adiponectin levels increase inflammation and oxidative stress, exacerbating nephropathy (30). We found that HFD-induced decrease in adiponectin level was up-regulated by Cinnamaldehyde treatment, suggesting a reno-protective effect. 

 The present study showed that HFD increases blood glucose, insulin, and HOMA-IR in C57BL/6 mice. These elevated levels are predictive tools for secondary-associated diseases such as diabetes, hyperinsulinemia, etc. Moreover, these elevations have been reported to elevate BUN, serum creatinine, and serum albumin, which are evident in renal dysfunction (31). In our study, HFD for 12 weeks caused elevated serum creatinine, serum albumin, and BUN levels. In addition, serum biomarkers and changes in the level of urinary creatinine and albumin excretion have been affected by HFD administration. Albuminuria is considered an early indicator of CKD (32), and higher levels are linked to cardiovascular diseases and kidney failure (33). CKD is also characterized by a decreased level of urine creatinine and an increase in the urinary albumin creatine ratio, which indicates the inability of renal cells to filter leftover materials from the urine. Numerous investigations have presented the link between obesity and kidney disease and are supported by changes in elimination by renal (34). Due to this, it is considered a helpful resource for identifying kidney disease (35). Treatment with Cinnamaldehyde exerts nephroprotection by attenuating HFD-induced changes in serum (BUN, blood glucose, HOMA-IR, serum creatinine, and serum albumin) and urinary (creatinine and albumin and urinary albumin creatinine ratio), thus improving renal function. 

Further, HFD administration for 12 weeks increased the triglycerides and total cholesterol. Also, it is reported that HFD significantly impacts TG and TC (24). Studies also reported that elevated levels of TC and TG continue to trigger the development of oxidative stress (36). In addition, earlier research has also stipulated that a rise in proinflammatory cytokines is associated with metabolic disorders like obesity (37). Secondly, **IL-1β, IL-6, TNF-α, and IL-18 are overexpressed in obesity, contributing to the development of nephropathy by promoting inflammation (**37**). Existing data suggest that IL-1β is directly involved in leptin expression and leads to hyperleptinemia (**38**); elevated levels of IL-6 have been linked to dysfunction of mesangial cells and tubular epithelial cells (**37**). Moreover, elevated levels of TNF-α stimulate the generation of collagen and extracellular matrix protein, and** IL-18 is a key mediator linking obesity to systemic inflammation and nephropathy (39). Elevated expression and urinary levels of IL-18 in obese individuals contribute to chronic inflammation and renal damage (40). We found that consumption of HFD significantly raised the levels of blood proinflammatory cytokines, TG, TC, and urinary proinflammatory IL-18. However, Cinnamaldehyde treatment markedly decreased elevated serum TG, TC, proinflammatory cytokines, and urinary proinflammatory IL-18. Ample evidence indicates the potential role of the NF-kB pathway through an increase in proinflammatory cytokines causing inflammation and organ damage (41). 

Our investigational study provides initial evidence that Cinnamaldehyde may reduce obesity-related nephropathy by decreasing the production of inflammatory cytokines, which play a crucial part in renal and adipocyte malfunction. 

We also assessed the anti-oxidant activity of Cinnamaldehyde. HFD reduced the level of anti-oxidants after 12 weeks. Oxidative stress inhibited mesangial cell activation and caused nephropathy, which is concorded with previous reports (42). A decreased anti-oxidant enzyme activity indicates loss of function in renal tissue, increased GRF, and glomerulosclerosis. The anti-oxidant enzymes maintain reactive oxygen species (ROS) under normal conditions. SOD, GPx, GST, and the non-enzymatic anti-oxidant GSH are anti-oxidant enzymes that counteract oxidative damage by neutralizing free radicals. Oxidative stress drastically alters proteins, cellular lipids, and DNA (43). The SOD converts superoxide radicals into hydrogen peroxide and oxygen, which are less reactive and less damaging to cells (44). GPx is responsible for leveraging GSH to reduce hydrogen peroxide and organic hydroperoxides to water and corresponding alcohol, respectively (45). A reduction in the level of GSH indicated a rise in oxidative stress and a decrease in GST activity, as the action required for GSH for GPX and GST. In our study, treatment with Cinnamaldehyde increased the levels of kidney anti-oxidants, indicating a decrease in ROS production, preventing renal damage, and causing nephroprotection.

Further, a transmembrane glycoprotein called KIM-1 (kidney injury molecule-1) is present in the proximal tubular cell (PTC). It is a key indicator of damage to the PTC (46). It is absent in the urine of humans and animals unless kidney injury is present. Notably, its levels elevate earlier than serum creatinine (47). Elevation of KIM-1 in the urine is a highly sensitive biomarker for assessing kidney injury in obese individuals (47). In the study, 12-week HFD administration caused an increase in the level of KIM-1 in urine, which signified nephropathy. This is in concurrence with previous reports (48). Overexpression of KIM-1in urine was suppressed by Cinnamaldehyde, which suggests the inhibition of inflammatory pathways. The association of KIM-1 and Cinnamaldehyde has not been reported so far. 

In response to structural damage, tubular cells release a glycoprotein known as neutrophil gelatinase-associated lipocalin (NGAL). NGAL is considered a sensitive biomarker for acute kidney injury. An increased NGAL in the urine occurs due to tubular lesions (49). In our study, we observed that HFD administration caused an increased NGAL level in urine, which may be correlated to inflammation and oxidative stress, causing tubular damage. Previous studies also reported urine NGAL and kidney damage (50). Cinnamaldehyde treatment prevented inflammation and oxidative stress, resulting in decreased NGAL levels in urine. Our study is unique as it highlights the role of Cinnamaldehyde in nephropathy associated with obesity.

Accumulating evidence also suggests the cross-link between obesity, hyperleptinemia, and type-IV collagen. Type-IV collagen is found in podocytes, proximal tubular cells, and the mesangial matrix. It is essential for preserving the kidney’s structural integrity (51). Studies showed that obesity and hyperleptinemia stimulate the formation of type-IV collagen, which in turn increases the extracellular matrix and causes glomerulosclerosis and renal fibrosis (51). In the existing study, HFD administration increased urine type-IV collagen, similar to previous reports (52). Cinnamaldehyde treatment showed a marked reduction of urine type-IV collagen. However, the result of our present study brought to light that Cinnamaldehyde has nephroprotective potential, which has not been reported so far.

Histopathological analyses of kidney tissue stained with H&E revealed glomerular inflammation (congested glomeruli), tubular injury, and tubular degeneration and necrosis (tubular cells swollen with vacuoles). There was also inflammatory infiltration in tubules and glomeruli after 12 weeks in HFD mice. However, Cinnamaldehyde treatment decreases HFD-induced alterations due to reduced levels of oxidative damage, proinflammatory cytokines, and adipokines, ultimately resulting in the prevention of nephropathy linked to obesity.

Obesity is an excessive dual energy state that reduces the activity of AMPK. The AMPK pathway is critical for energy balance and cellular metabolism (53). Adenosine-5-triphosphate (ATP) production drops, and ATP demand rises during the metabolic phase, which triggers the AMPK system to work harder. Furthermore, the enzyme acetyl-CoA carboxylase (ACC) regulates the synthesis of malonyl-CoA, which is essential for the production of fatty acids (FAs) and the regulation of hepatic mitochondrial FA oxidation (53). The activation of AMPK regulates the phosphorylation of ACC. Studies documented that obesity reduced the activity of AMPK, which causes renal tissue to undergo proliferative enlargement and accumulation of the matrix of renal tissue (53). Immunohistochemistry studies of kidney tissues showed positive staining for activated AMPK in the normal control group. Conversely, though, 12 weeks of HFD revealed a reduction in the quantity of immune-stained AMPK-positive cells, indicating dysfunction of AMPK in kidney tissue. However, treatment with Cinnamaldehyde significantly increased the number of positive cells, indicating the initiation of the AMPK signaling pathway and improving renal function. This is in concurrence with the previous study (54). 

Chronic inflammation is linked to obesity, generating proinflammatory cytokines such as TNF-α, IL-6, IL-1β, and IL-18 that activate NF-kB (39). NF-kB is overexpressed in obesity and plays a part in the emergence of obesity-associated nephropathy (41). In our study, we observed increased levels of TNF-α, IL-6, IL-1β, and IL-18 by feeding HFD. Immunohistochemistry studies of kidney tissue showed positive staining for the stimulation of NF-kB in the HFD group, indicating increased inflammation due to obesity causing renal structural dysfunction. Cinnamaldehyde treatment significantly reduced NF-kB-positive staining cells, inhibiting the inflammatory pathway and showing reno-protective potential. Previous studies also reported that inflammation was suppressed by alteration in NF-kB activity (55). 

Lastly, it was witnessed that Cinnamaldehyde at the dose of 40 mg/kg was more impactful in significantly lessening body weight, blood glucose, serum insulin, IR, serum creatinine, serum albumin, BUN, urine albumin, UACR, TG, TC, serum leptin, serum adiponectin, TNF-α, IL-6, IL-1β, and IL-18. KIM-1, type-IV collagen, NGAL, SOD, GPx, GST, and GSH and elevating urinary creatinine as compared with Cinnamaldehyde 20 mg/kg in 12 weeks HFD treated mice. Histopathological and immunohistochemical examinations validated the outcomes after administering Cinnamaldehyde at 40 mg/kg. Hence, the present study demonstrated that Cinnamaldehyde treatment (40 mg/kg) effectively ameliorated HFD-induced obesity-associated nephropathy. 

**Table 1:** *In silico *study docking score of Cinnamaldehyde and Orlistat with the active binding site of AMPK and NF-kB in C57BL/6 mice

Sr. No.	Ligand	AMPK (PDB ID: 4CFF)	NF-kB (PDB ID:40T9)
1.	Cinnamaldehyde	-4.4	-3.8
2.	Orlistat	-5.5	-4.6

**Table 2:** Effect of Cinnamaldehyde on body weight, BMI, food intake, kidney weight, and kidney/body weight in HFD-induced obesity-associated nephropathy in C57BL/6 mice

Groups	Body weight (gm)	BMI	Food intake (gm)	Kidney weight (gm)	Kidney/body weight
Group I / Vehicle Group	29.05±0.34	0.41±0.005	3.74±0.04	0.32±0.01	0.011±0.000
Group II / HFD group	44.35±0.48^***^	0.60±0.006^***^	3.54±0.03^ ns^	0.55±0.01^***^	0.012±0.000^ ns^
Group III / HFD + CA (20 mg/kg, PO)	43.35±0.61^###^	0.61±0.008^ ns^	3.68±0.04^ ns^	0.52±0.01^ns^	0.012±0.000^ ns^
Group IV / HFD + CA (40 mg/kg, PO)	36.35±0.54^£££^	0.52±0.008^£££^	3.63±0.04^ ns^	0.41±0.01^£££^	0.011±0.000^ ns^
Group V / HFD + Orli (10 mg/kg, PO	35.35±0.56^$$$^	0.51±0.008^$$$^	3.62±0.04^ ns^	0.38±0.01^$$$^	0.010±0.000^ ns^
Group VI / CA Perse (40 mg/kg, PO)	28.05±0.36	0.39±0.005	3.54±0.03	0.33±0.01	0.012±0.000^ ns^

Results are represented as mean ± SEM (n=10) and analyzed by one-way ANOVA followed by Tukey's multiple comparisons test

HFD: High fat diet; CA: Cinnamaldehyde. ***P<0.001 Vehicle group vs HFD group; ### *P*<0.001 HFD group vs HFD + CA (20 mg/kg, PO); £££ *P*<0.001 HFD group vs HFD + CA (40 mg/kg, PO); $$$ *P*<0.001 HFD group vs HFD + Orli (10 mg/kg, PO); ns *P*>0.05 Vehicle group vs HFD group, HFD group vs HFD + CA (20 mg/kg, PO), HFD group vs HFD + CA (40 mg/kg, PO), HFD group vs HFD + Orli (10 mg/kg, PO)

**Table 3:** Effect of Cinnamaldehyde on blood glucose, serum insulin, and HOMA-IR in HFD-induced obesity-associated nephropathy in C57BL/6 mice

Groups	Blood glucose (mg/dl)	Serum insulin (uIU/ml)	HOMA-IR
Group I / Vehicle group	107.6± 3.24	3.34±0.30	0.90±0.10
Group II / HFD group	189.1±3.18^***^	6.76±0.19^***^	3.16±0.11^***^
Group III / HFD + CA (20 mg/kg, PO)	163.8±2.92^###^	5.64±0.23^#^	2.29±0.12^###^
Group IV / HFD + CA (40 mg/kg, PO)	136.5±3.18^£££^	4.35±0.29^£££^	1.47±0.11^£££^
Group V / HFD + Orli (10 mg/kg, PO	112.2±3.77^$$$^	3.19±0.22^$$$^	0.88±0.07^$$$^
Group VI / CA *Perse *(40 mg/kg, PO)	104.6±3.33	3.25±0.29	0.85±0.09

Results are represented as mean ± SEM (n=10) and analyzed by one-way ANOVA followed by Tukey's multiple range test.

HFD: High fat diet; CA: Cinnamaldehyde. IU/ml: International units’ activity per millilitre

***P<0.001 Vehicle group vs HFD group ; #*P*<0.05, ### *P*<0.001 HFD group vs HFD + CA (20 mg/kg, PO); £££ *P*<0.001 HFD group vs HFD + CA (40 mg/kg, PO); $$$ *P*<0.001 HFD group vs HFD + Orli (10 mg/kg, PO)

**Table 4:** Effect of Cinnamaldehyde on serum total cholesterol and serum total triglyceride in HFD-induced obesity-associated nephropathy in C57BL/6 mice

Groups	Serum total cholesterol (mg/dl)	Serum total triglycerides (mg/dl)
Group I / Vehicle group	117.5±2.59	78.06±4.24
Group II / HFD group	162.8±3.22^***^	175.5±4.73^***^
Group III / HFD + CA (20 mg/kg, PO)	148.7±3.25^#^	152.8±3.40^##^
Group IV / HFD + CA (40 mg/kg, PO)	121.4±3.16^£££^	131.0±3.94^£££^
Group V/ HFD + Orli (10 mg/kg, PO	114.1±3.18^$$$^	77.11±3.96^$$$^
Group VI / CA *Perse *(40 mg/kg, PO)	114.5±2.62	77.09±4.30

Results are represented as mean ± SEM (n=10) and analyzed by one-way ANOVA followed by Tukey's multiple range test.

HFD: High fat diet; CA: Cinnamaldehyde. ***P<0.001 Vehicle group vs HFD group; # *P*<0.05, ##*P*<0.01 HFD group vs HFD + CA (20 mg/kg, PO); £££ *P*<0.001 HFD group vs HFD + CA (40 mg/kg, PO); $$$ *P*<0.001 HFD group vs HFD + Orli (10 mg/kg, PO)

**Table 5:** Effects of Cinnamaldehyde on BUN, serum creatinine, and serum albumin in HFD-induced obesity-associated nephropathy in C57BL/6 mice

Groups	BUN (mg/dl)	Serum creatinine (mg/dl)	Serum albumin (g/dl)
Group I / Vehicle group	19.41±1.20	0.37±0.01	4.39±0.27
Group II / HFD group	43.01±1.18^***^	1.65±0.07^***^	8.88±0.23^***^
Group III / HFD + CA (20 mg/kg, PO)	37.32±1.32^##^	1.45±0.06^#^	7.60±0.30^#^
Group IV / HFD + CA (40 mg/kg, PO)	30.52±1.03^£££^	1.16±0.04^£££^	7.34±0.28^££^
Group V / HFD + Orli (10 mg/kg, PO	25.65±1.00^$$$^	0.91±0.03^$$$^	5.49±0.29^$$$^
Group VI / CA *Perse *(40 mg/kg, PO)	21.95±0.86	0.41±0.01	4.44±0.24

Results are represented as mean ± SEM (n=10) and analyzed using one-way ANOVA followed by Tukey's multiple range test.

HFD: High fat diet, BUN: Blood urea nitrogen; CA: Cinnamaldehyde. g/dl= Gm per decilitre

***P<0.001 Vehicle group vs HFD group. # *P*<0.05, ##*P*<0.01 HFD group vs HFD + CA (20 mg/kg, PO); ££ *P*<0.01 and £££ *P*<0.001 HFD group vs HFD + CA (40 mg/kg, PO); $$$ *P*<0.001 HFD group vs HFD + Orli (10 mg/kg, PO)

## Conclusion

The present study explored the role of Cinnamaldehyde in obesity-associated nephropathy induced by HFD in the preclinical rodent model. We investigated the possible pathways involved in the nephroprotective effect of Cinnamaldehyde. We found that Cinnamaldehyde treatment (40 mg/kg, PO) significantly decreased changes like hyperleptinemia, insulin resistance, inflammation, oxidative stress, and adipocyte hypertrophy in C57BL/6 mice, and results were comparable with standard anti-obesity drug orlistat (10 mg/kg, PO). In addition, Cinnamaldehyde modulated AMPK and NF-kB pathways to arrest the progression of obesity and associated nephropathy. These findings suggest the potential of Cinnamaldehyde to mitigate obesity and related nephropathy. We propose that Cinnamaldehyde can be considered as a promising therapeutic molecule against HFD-induced obesity and related diseases like nephropathy. The potential of Cinnamaldehyde may be probed further to establish its clinical benefits. 

## Declaration

We have not used any AI tools or technologies to prepare this manuscript.
